# Endomicrobiome of *in vitro* and natural plants deciphering the endophytes-associated secondary metabolite biosynthesis in *Picrorhiza kurrooa*, a Himalayan medicinal herb

**DOI:** 10.1128/spectrum.02279-23

**Published:** 2023-10-09

**Authors:** Anish Tamang, Mohit Swarnkar, Pawan Kumar, Dinesh Kumar, Shiv Shanker Pandey, Vipin Hallan

**Affiliations:** 1 Biotechnology Division, CSIR-Institute of Himalayan Bioresource Technology (IHBT), Palampur, Himachal Pradesh, India; 2 Academy of Scientific and Innovative Research (AcSIR), Ghaziabad- 201002, India; 3 Chemical Technology Division, CSIR-Institute of Himalayan Bioresource Technology, Palampur, Himachal Pradesh, India; Connecticut Agricultural Experiment Station, New Haven, Connecticut, USA

**Keywords:** Endomicrobiome, plant microbiota, endophytes, secondary metabolites, picrosides, next-generation DNA sequencing

## Abstract

*Picrorhiza kurrooa* an endangered high-altitude Himalayan medicinal herb, is used as a potent hepatoprotective due to the presence of various secondary metabolites, with picrosides being the most bioactive. *In-vitro* propagation is used as a sustainable strategy for its conservation and cultivation. The *in-vitro* propagation produces *P. kurrooa* plants (Tc) in mass, but these plants have reduced secondary metabolites (especially picrosides) compared to plants growing in their natural habitats (Wt). Therefore, considering the well-established role of microbes in secondary metabolite biosynthesis, in this study, endomicrobiome of *P. kurroa* plants (Tc and Wt) was explored. Using high-throughput DNA-sequencing, the endophytic communities associated with leaves, roots, and rhizomes of Wt and Tc plants were characterized. Diversity analysis revealed a loss of diversity during *in-vitro* propagation, and the abundant phyla were Proteobacteria, Bacteroidetes, Parcubacteria (OD1), Firmicutes, and Verrucomicrobia. Besides, the presence of distinct genera specific to different parts of Wt plants was also revealed. Quantification of secondary metabolites demonstrated the reduced accumulation of picrosides and intermediates of picroside biosynthesis in the Tc plants compared to Wt plants. Host-secondary metabolite production was positively correlated to microbial community abundance, suggesting a dynamic interplay of host-endomicrobiota interaction. Predictive functional analysis revealed the abundance of enzymes of secondary metabolite biosynthesis (especially MVA/MEP and phenylpropanoid/shikimate pathway involved in picrosides biosynthesis) in the associated-endophytic community with predominance in roots and rhizomes of Wt plants. This investigation provides novel insight into the change in the endomicrobiome of Wt and Tc plants and their correlation to the biosynthesis of secondary metabolites, and that needs to be considered for cultivation practices.

**IMPORTANCE:**

*Picrorhiza kurrooa* is a major source of picrosides, potent hepatoprotective molecules. Due to the ever-increasing demands, overexploitation has caused an extensive decline in its population in the wild and placed it in the endangered plants' category. At present plant *in-vitro* systems are widely used for the sustainable generation of *P. kurrooa* plants, and also for the conservation of other commercially important, rare, endangered, and threatened plant species. Furthermore, the *in-vitro*-generated plants had reduced content of therapeutic secondary metabolites compared to their wild counterparts, and the reason behind, not well-explored. Here, we revealed the loss of plant-associated endophytic communities during *in-vitro* propagation of *P. kurrooa* plants which also correlated to in-planta secondary metabolite biosynthesis. Therefore, this study emphasized to consider the essential role of plant-associated endophytic communities in *in-vitro* practices which may be the possible reason for reduced secondary metabolites in *in-vitro* plants.

## INTRODUCTION

*Picrorhiza kurrooa* Royle ex Benth. is a perennial medicinal herb predominantly found in the Himalayas at an altitude of 3,000–5,000 m above mean sea level and belongs to the Plantaginaceae family (1). Medicinal properties of *P. kurrooa* plant are due to the presence of biologically active secondary metabolites, Picroside-I (P-I) and Picroside-II (P-II) (iridoid glycosides), which are known to be differentially present in different parts of the plant and have potent hepatoprotective properties (2). *P. kurrooa* is also used to treat jaundice, malaria, asthma, and liver disorder (3) and is commercially available in many drug formulations like Katuki, Livocare, Livomap, Livplus, and so on. *P. kurrooa* has been listed by the International Union for Conservation of Nature and Natural Resources as an endangered species (4). Due to its immense commercial value, and overexploitation of this herb, there is an urgent need for novel strategies for conserving this plant along with a supply of consistent quality raw material without compromising the levels of major secondary metabolites. Therefore, several attempts have been made for the cultivation and conservation of *P. kurrooa* plant. Mass propagation of *P. kurrooa* shoots through plant tissue culture (*in vitro* culture) is a most prominent strategy for its cultivation. However, *in vitro* micro-propagated *P. kurrooa* plants had lower yields of picrosides than the plants of natural habitats (5, 6) and the reason behind the reduced content of picrosides in *in vitro* generated plants is relatively less explored.

Plants are not just solitary organisms but exist in complex communities with diverse microorganisms. These microbial communities colonize naturally in different parts of a plant including stem, leaves, roots, flowers, fruits, and seeds. These microorganisms, collectively known as the plant microbiome, include both beneficial and pathogenic microbes that reside both inside and outside the plant tissues and play critical roles in the health and well-being of their host plants. Microbes residing inside the plant tissues are considered endophytes that build the plant’s endo-microbiome and are found to play a significant role in plant health (7). Recent advances in DNA sequencing and other molecular techniques enabled researchers to explore the diversity and function of the plant microbiome in unprecedented detail. As a result, the fascinating ways of interaction of microorganisms with plants, contributing to their growth, development, and response to environmental stresses are being uncovered. The study of plant microbiomes has significant implications for agriculture, as understanding the complex relationships between plants and their microbial partners can help us to develop more sustainable, productive, and eco-friendly agricultural practices. With the advent of high-throughput sequencing technologies over the last few decades, host-associated microbiomes have been further investigated (8 – 12). These techniques have been extensively utilized to investigate the human microbiome (13 – 19) and have illustrated the association of gut microbial structure with host nutrition (20), diseases (21 – 23), and also host behavior (24, 25). Similarly, the functions of plant microbiome are also found crucial for plant well-being and fitness, with many reports suggesting the diverse nature of microbial communities colonizing plants with various functionality, with more cell numbers than the host itself (7, 26). Plant microbiomes are known to demonstrate growth-promoting effects (27 – 31), resilient against pathogens (32, 33), and provide an overall positive effect on plant fitness (34, 35). Moreover, along with the role of the microbiome in pathogen resistance and overall fitness, there have been many investigations revealing the significant impact of the microbiome on secondary metabolite biosynthesis and the accumulation of medicinal plants (36 – 42). Medicinal plants constitute a wide range of pharmacologically important secondary metabolites and also harbor endophytes producing bioactive substances. Several studies have suggested the colonization of bacteria in the internal tissues of plants, and these endophytic bacteria play a significant role in the modulation of plants’ metabolite synthesis (43). Therefore, understanding the role of endophytes associated with a particular plant is critical to understand metabolite synthesis.

Therefore, this study aimed to decipher the endophytic bacterial diversity in the Wild (Wt) and tissue cultured *in vitro* propagated (Tc) *P. kurrooa* plants. The diversity was analyzed in different tissues of both types of plants using the Illumina MiSeq sequencing platform. The present investigation intended to provide insight into the change/shift in plant endomicrobiome during *in vitro* propagation. We also attempted to understand the secondary metabolite accumulation in both Wt and Tc plants and the potential role of plant endomicrobiome in this modulation. Here, we put forward the first report of the endomicrobiota of *P. kurrooa* and the dynamic role of endophytes in host secondary metabolism, which will add novel insight into understanding the interaction of this medicinal plant with beneficial endophytic microbial community.

## RESULTS

### Difference in taxa abundance of bacterial endophytes in different parts of wild and tissue-cultured plants

**Fig 1:**
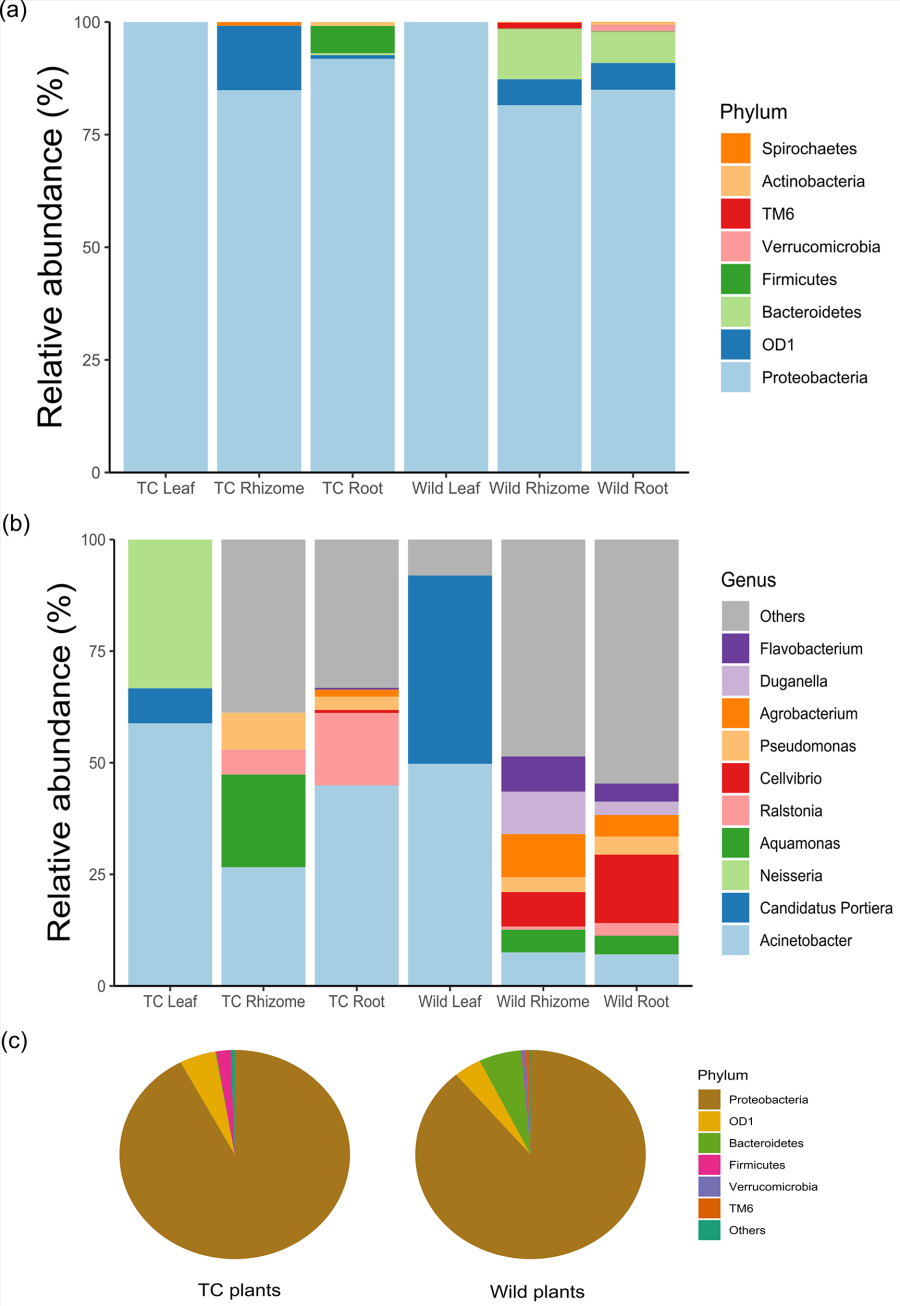
Relative abundance of bacterial taxa in different anatomical parts of Wt and Tc *Picrorhiza kurrooa* plants at (**a**) phylum and (**b**) genus level. Pie chart showing abundance of Wt and Tc plant at (**c**) phylum level.

The taxonomic classification of the sequence revealed 423 operational taxonomic units (OTUs) at ≥97% similarity and was grouped into eight phyla. Abundance, accounting for most taxa across different vegetative parts (roots, rhizomes, and leaves) of the plant, was analyzed. The abundant phyla in all the samples were Proteobacteria (85.6%), followed by Bacteroidetes (6.4%) and Firmicutes (1.5%) phlya, namely OD1 (Parcubacteria), Verrucomicrobia, Actinobacteria, Spirochaetes, and TM6 (candidate phylum Dependentiae) were also present (Fig. 1a; Table S1). At the genus level, overall bacterial diversity was higher in Wt plants than in Tc plants, suggesting a loss of endophytic diversity during the process of *in vitro* propagation (Fig. 1b; Table S2).

**Fig 2:**
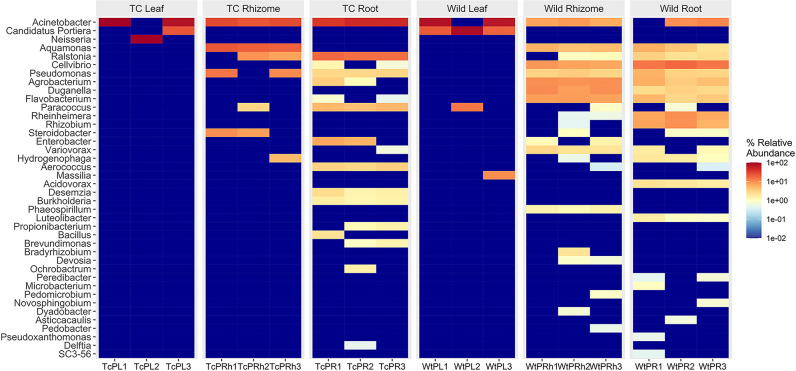
Heat map showing relative abundance of different genera of bacteria in different anatomical parts of the Wt and Tc plants.

**Fig 3:**
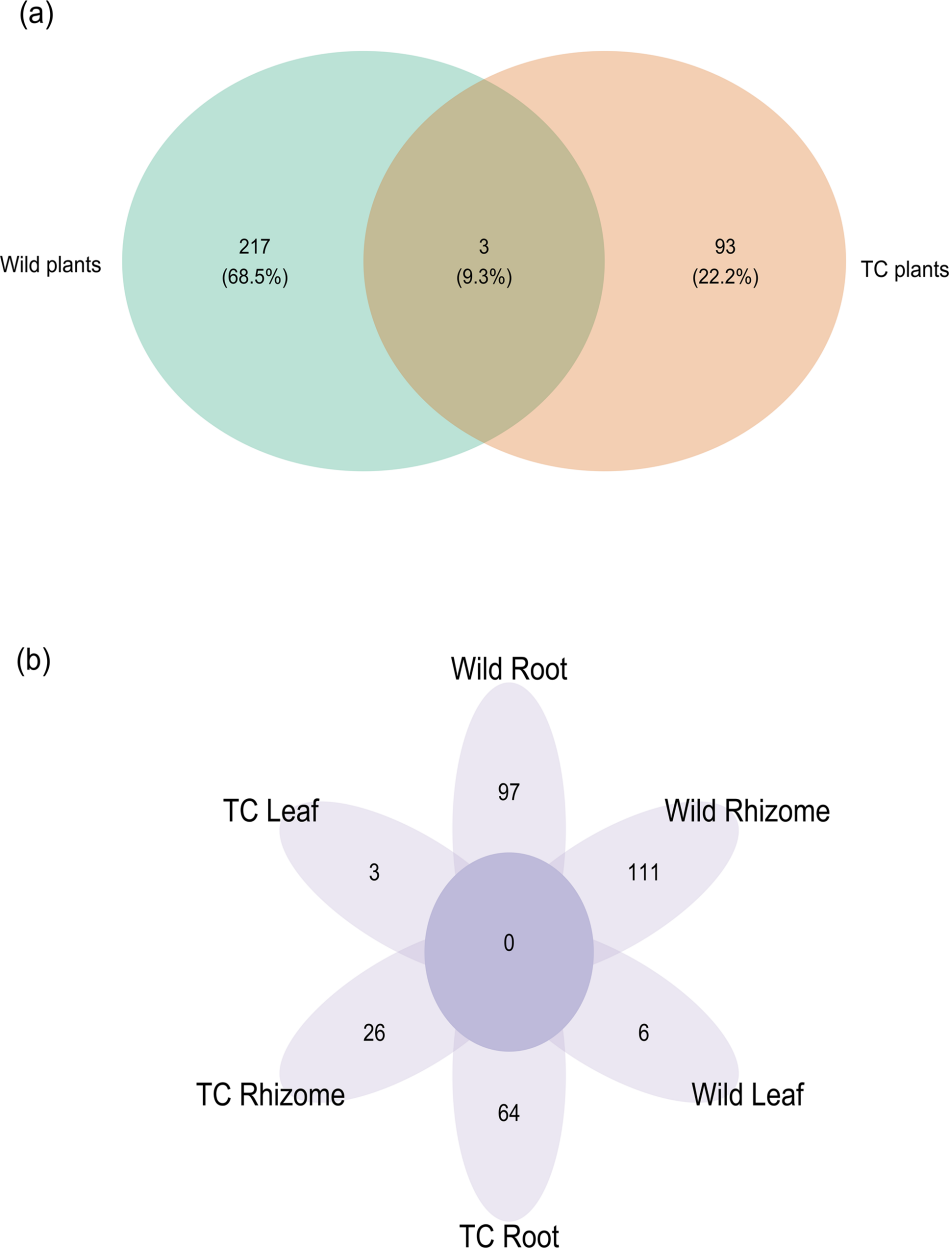
Venn diagram depicting the (**a**) shared and distinct OTU in Wt and Tc plants. (**b**) OTUs present uniquely in different anatomical parts of Wt and Tc *P. kurrooa* plants.

Besides, leaves of Wt plants had endophytes belonging to distinct genera including *Paracoccus*, *Massilia*, and *Arsenophonus* which were absent in leaves of Tc plants. Similarly, for root tissue, the distinct genera that were present only in roots of Wt plants were *Acidovorax*, *Steroidobacter*, *Microbacterium*, *Pseudoxanthomonas*, *Novosphingobium*, *Aquamonas*, *Peredibacter*, *Asticcacaulis*, *Legionella*, *Steroidobacter*, *Hydrogenophaga*, *Duganella*, *Rhizobium*, *Rheinheimera*, and *Luteolibacter*, whereas for Wt rhizome tissue they were *Cellvibrio*, *Agrobacterium*, *Rhizobium*, *Flavobacterium*, *Duganella*, *Enterobacter*, *Variovorax*, *Rheinheimera*, *Bradyrhizobium*, *Phaeospirillum*, *Aerococcus*, *Pedomicrobium*, *Devosia*, *Phaespirillum*, *Spirochaeta*, *Dyadobacter*, *and Pedobacter* (Fig. 2). Predominance of Acinetobacter was also observed in leaf (61.7%), root (45.1%), and rhizome (26.59%) tissues of Tc plants, making it the dominant genus colonizing the Tc plant. The number of distinct OTUs was also higher in Wt plants (217) compared to Tc plants (44) (Fig. 3a). Besides, in Wt plants 6 OTUs in leaves, 97 OTUs in roots, and 111 OTUs in rhizomes were unique, whereas in the case of Tc plant 3 OTUs in leaves, 26 OTUs in roots, and 64 OTUs in rhizomes were unique Fig. 3b.

### Diversity indices across different parts of Wt and Tc plants

**Fig 4:**
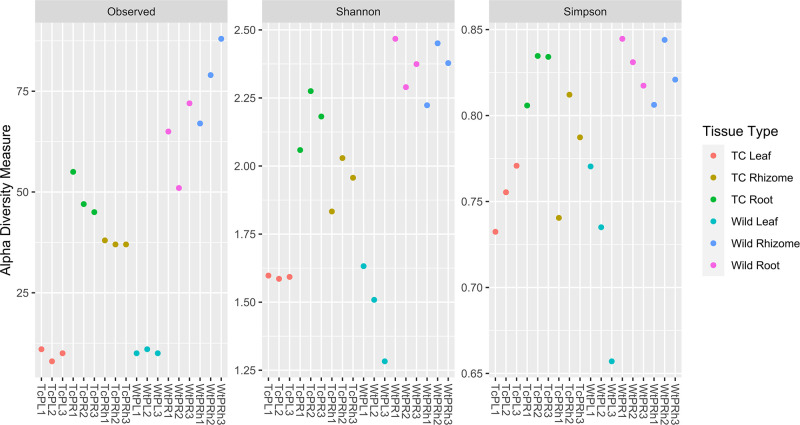
Alpha diversity based on observed number of species, Shannon and Simpson indexes. The *Y*-axis indicates the values for the corresponding index. The colored spots represent different anatomical parts of the plants. TC Leaf: Replicates (TcPL1, TcPL2, and TcPL3); TC Root: Replicates (TcPR1, TcPR3, and TcPR3), TC Rhizome: Replicates (TcPRh1, TcPRh2, and TcPRh3); Wild Leaf: Replicates (WtPL1, WtPL3, and WtPL3); Wild Root: Replicates (WtPR1, WtPR2, and WtPR3); and Wild Rhizome: Replicates (WtPRh1, WtPRh2, and WtPRh3).

Microbial alpha diversity indices such as Observed, Shannon, and Simpson were analyzed. The observed species index, which depicts the total number of species in a sample, indicated comparatively more bacterial richness in the rhizome and root of Wt plants than in Tc plants. Simpson and Shannon’s indexes also reflected this, which depict microbial abundance (evenness) and richness, respectively. However, both Wt and Tc leaf tissues observed similar diversity index values (Fig. 4). ANOVA was utilized to determine the significance level for Observed (*P* < 0.001, *F* = 21.34), Shannon (*P* < 0.001, *F* = 35.14), and Simpson (*P* < 0.001, *F* = 24.46) indexes, whereas in case of non-parametric Kruskal-Wallis test was applied with statistical significance level for Observed (*P* = 0.007, *F* = 15.67), Shannon (*P* = 0.007, *F* = 15.83), and Simpson (*P* = 0.007, *F* = 15.87) (Fig. 4).

**Fig 5:**
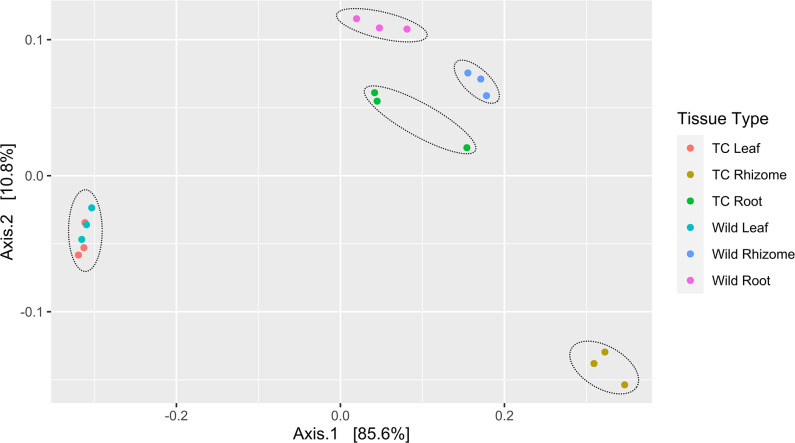
Beta diversity analysis, two-dimensional scatter plots generated using PCoA based on UniFrac distance metric. Samples corresponding to six groups were plotted as colored spots.

The beta diversity analysis revealed that distinct clusters of microbial community were observed wherein the roots and rhizomes of the Wt plants were placed apart from the roots and rhizomes of the Tc plants. Leaves of the Wt and Tc plants formed a closed cluster, as this similarity also corroborates the leaf sample’s alpha diversity indexes. Principal Coordinate Analysis (PcoA) was utilized to depict the beta diversity, with Bray-Curtis dissimilarity index, and statistical significance was computed using PERMANOVA (*F* = 22.33, *R* ^2^ = 0.9, *P* = 0.001) and ANOSIM (*R* ^2^ = 0.99, *P* < 0.001) (Fig. 5).

### Determination of shared, difference in abundance and core endophytes for microbial community

**Fig 6:**
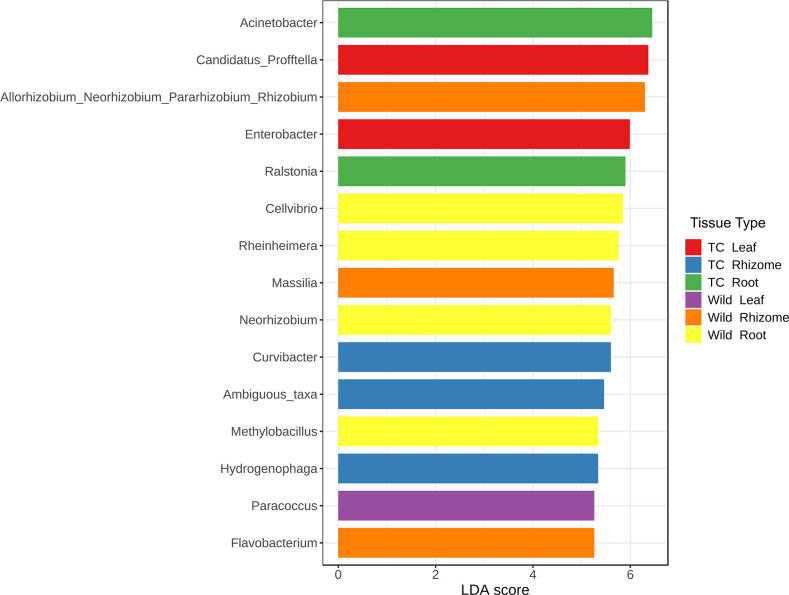
Differentially abundant bacterial taxa found in *Picrorhiza kurrooa*. Linear discriminant analysis (LDA) effect size (LEfSe) comparison of relative abundance. Horizontal bars represent the effect size for each taxon. LDA score cutoff of 2 was used.

**Fig 7:**
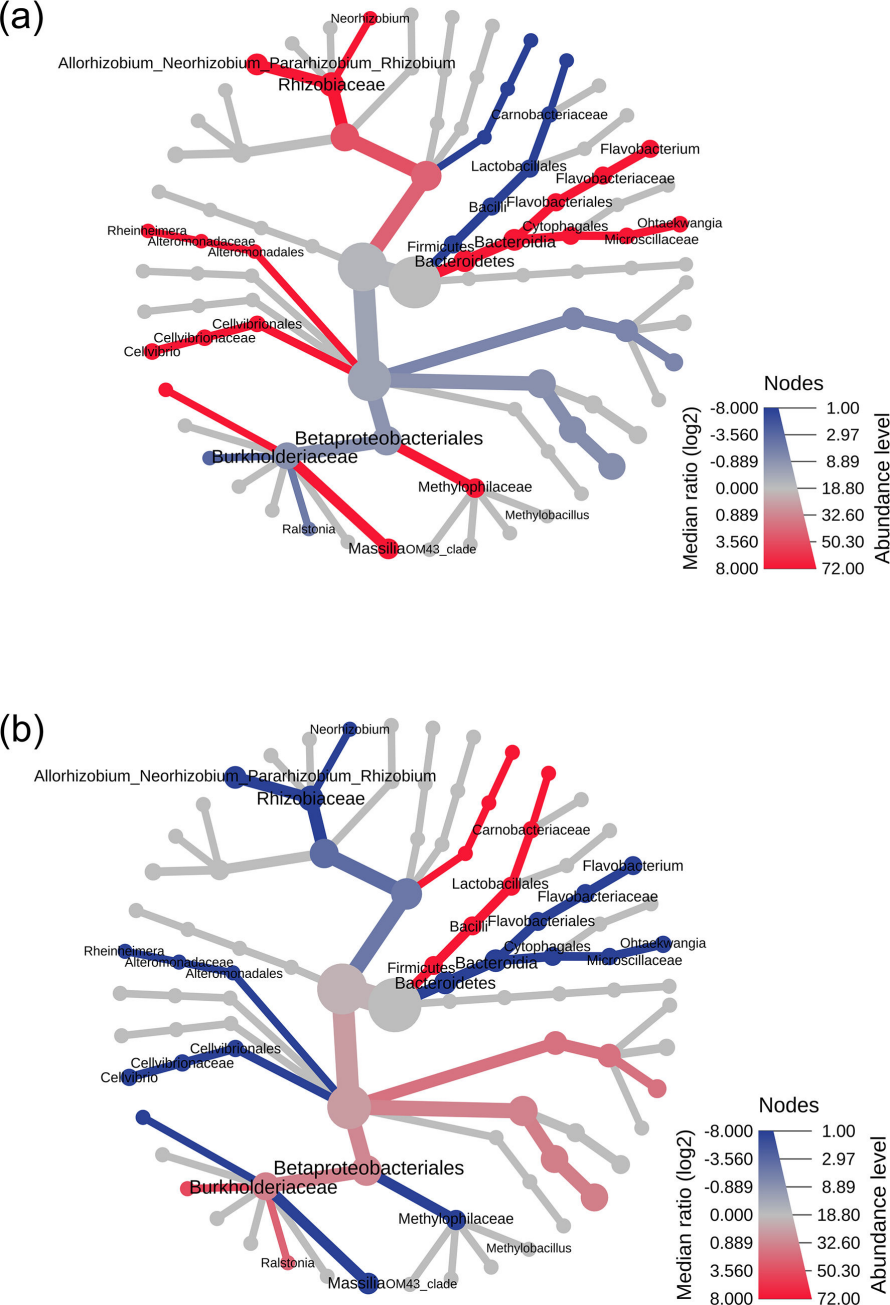
Heat tree illustrates the taxonomic differences between two comparisons (**a**) Wt versus Tc plants and (**b**) Tc versus Wt plants. The size of node, edge, color gradient, and label are based on the log_2_ ratio of median abundance. The blue and red colors indicate the higher and lower abundance of particular taxa, respectively.

LefSe analysis revealed taxa with significant abundance in different tissues of Wt and Tc plants, and it also provides insight into different taxa as potential biomarkers in various plant tissue. *Paracoccus* was abundant in Wt leaf tissues, with *Cellvibrio*, *Rheinheimera*, *Neorhizobium*, and *Methylobacillus* being significantly abundant in Wt root tissues, whereas in the Wt rhizome *Allorhizobium*/*Neorhizobium*/*Pararhizobium*/*Rhizobium* and *Flavobacterium* was significantly abundant. The abundant taxa in the Tc plant were *Candidatus*, *Enterobacter* in leaf tissue, *Acinetobacter* and *Ralstonia* in the root, and *Curvibacter* and *Hydrogenophaga* in the rhizome tissues (Fig. 6; Table S3). Heat trees analysis was performed to compute the differential taxa between the Wt and Tc plants. The coloring depicts the significant differences between the median proportion reads for samples from both plants, determined using the Wilcox rank-sum test. In the heart tree, the color intensity is relative to the log_2_ ratio of the difference in median proportions. The red taxa represent enrichment in the particular plant, wherein Wt plants had an abundance of whole clad of phyla Bacteroidetes with genera *Flavobacterium* and *Ohtaekwangia*. Compared to Tc, other abundant genera found in the Wt plant included *Methylobacillus*, *Cellvibrio*, *Rheinheimera*, *Rhizobium*, *Neorhizobium*, and *Massilia* (Fig. 7).

**Fig 8:**
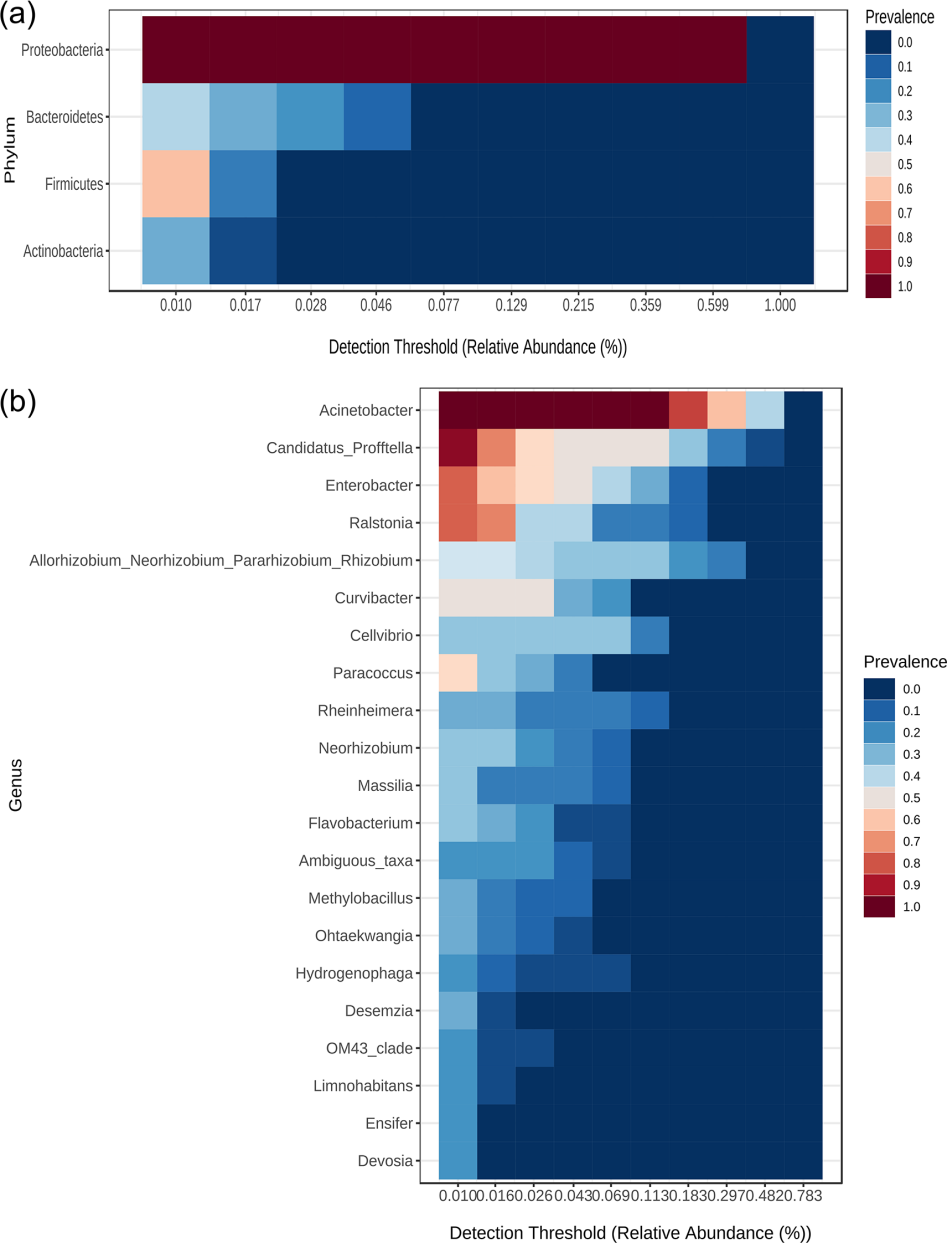
Core microbiota of endophytes in different anatomical parts of *Picrorhiza kurrooa* at (**a**) phylum level and (**b**) genus level. Core microbiome analysis is adopted from core functions of R package microbiome. The result is depicted in heat map form of core taxa where *Y*-axis represents the prevalence of core taxa across the detection threshold (relative abundance) range on *X*-axis.

The core taxa or features that remain unchanged in composition across the endophytic bacterial community were identified using Core microbiome analysis adopted from core function in R package microbiome. Variations in the abundance (OTU counts) at the genus level of the bacterial taxa were revealed by computing the core microbiome of different tissues of Wt and Tc plants. The heat map depicts the core taxa where the *Y*-axis represents the prevalence level of core features across the detection threshold (relative abundance) range on the *X*-axis. The result showed that *Acinetobacter* as the most prevalent taxa across tissues of both the plants, followed by *Enterobacter*, *Candidatus Profftella*, *and Ralstonia* with a prevalence level of more than 0.6 (Fig. 8; Table S4).

### Secondary metabolite profiling and its association with the microbial community of *Picrorhiza kurrooa*

**Fig 9:**
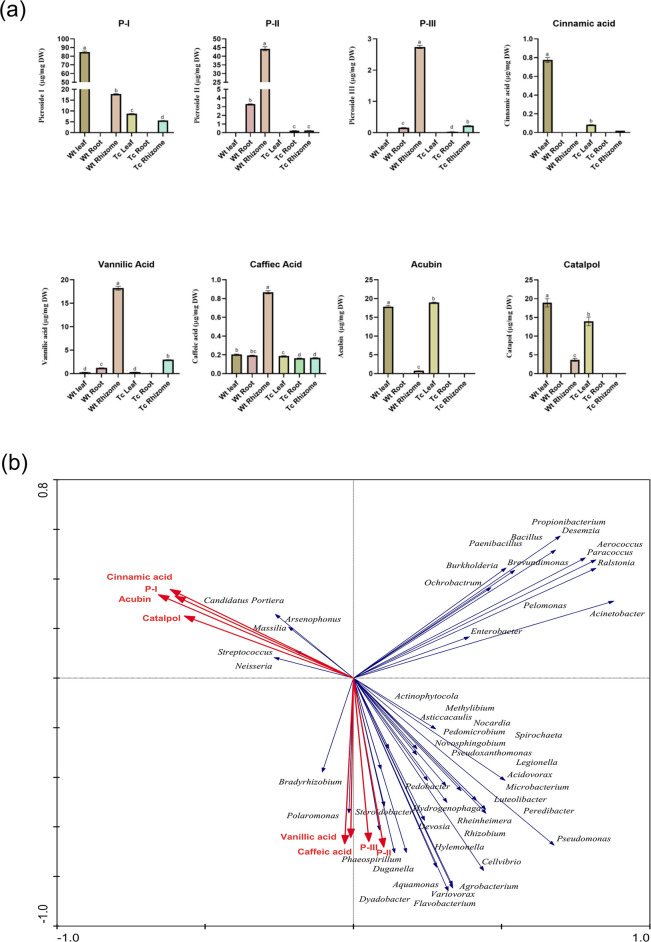
(a) UHPLC-PDA secondary metabolite analysis of leaf, root and rhizome of Wt (Wild) and Tc (Tissue cultured *in vitro* propagated) plants. (b) Redundancy analysis (RDA) depicting the correlation between bacterial community structure at genus level and the content of P-I, P-II, P-III, cinnamic acid, vanillic acid, caffeic acid, acubin, and catalpol.

Profiling of secondary metabolites, including Picrosides (P-I, P-II, and P-III) and their immediate precursors (Cinnamic acid, Vanillic acid, Caffeic acid, Acubin, and Catalpol), were done using UHPLC-PDA, revealed that the picroside accumulation was tissue specific. Higher content of picrosides was observed in different tissues of Wt plants in comparison to Tc plants. The content of Picroside-I (P-I) was found to be 9.6-fold and 3.6-fold higher in leaf and rhizome tissues, respectively, in comparison to Tc plant tissues. Moreover, P-I content was not detected in the root tissues of both Wt and Tc plants. Although in the case of P-II and P-III, both were not detected in leaf tissues of both Wt and Tc plants. However, the content of P-II was 13.9-fold and 180-fold higher in root and rhizome tissues of Wt plants, respectively, when compared to Tc plants. Similarly, the accumulation of P-III was higher in roots (5.7-fold) and rhizomes (12.5-fold) of Wt plants in comparison to their Tc plants counterparts. Further, the immediate precursors of picrosides also followed a similar trend, with Vanillic acid having 15.02-fold higher accumulation in root tissues and 6.14-fold in rhizome tissues, with no significant difference in leaf tissues. The content of Cinnamic acid was 9.2-fold higher in Wt leaf tissue, while its content in root and rhizome tissue was not detected. Caffeic acid content was also significantly higher (5.14-fold) in rhizome tissues of Wt plants, whereas the leaf and root tissues showed less variation in their content in Wt and Tc plants. Interestingly, the Acubin content was similar in leaf tissues of Wt and Tc plants, while it was not detected in root and rhizome of Tc plants. In contrast, it was detected in the rhizome of Wt plants. Accumulation of Catalpol was higher in leaves (1.3-fold) and rhizomes (3.66-fold) of Wt plants than that of Tc plants (Fig. 9a; Table S5).

Redundancy analysis (RDA) was performed based on the obtained endophytic bacterial community and the data of targeted secondary metabolite profiling of the processed samples to understand further the possibility of correlation of plant-associated bacterial endophytes and secondary metabolite biosynthesis of host plants. RDA reflected the endophytic bacterial community and secondary metabolites (mainly focusing on picrosides and their precursors) of plant samples on the same two-dimensional ordination map to describe the relationship between the endophytic community and secondary metabolites. Interestingly, P-I, and its immediate precursors Cinnamic acid, Acubin, and Catalpol showed a close correlation with bacterial community genera: *Massilia*, *Candidatus Portiera*, *Neisseria*, *Streptococcus*, and *Arsenophonus*. Although P-II, its precursor vanillic acid, caffeic acid, and P-III accumulation were closely co-related to the presence of *Bradyrhizobium*, *Polaromonas*, *Steroidobacter*, *Phaeospirillum*, and *Duganella* which were specifically present and/or abundant in tissues of Wt plants (Fig. 9b).

### Potential functional analysis of endophytic bacterial community

**Fig 10:**
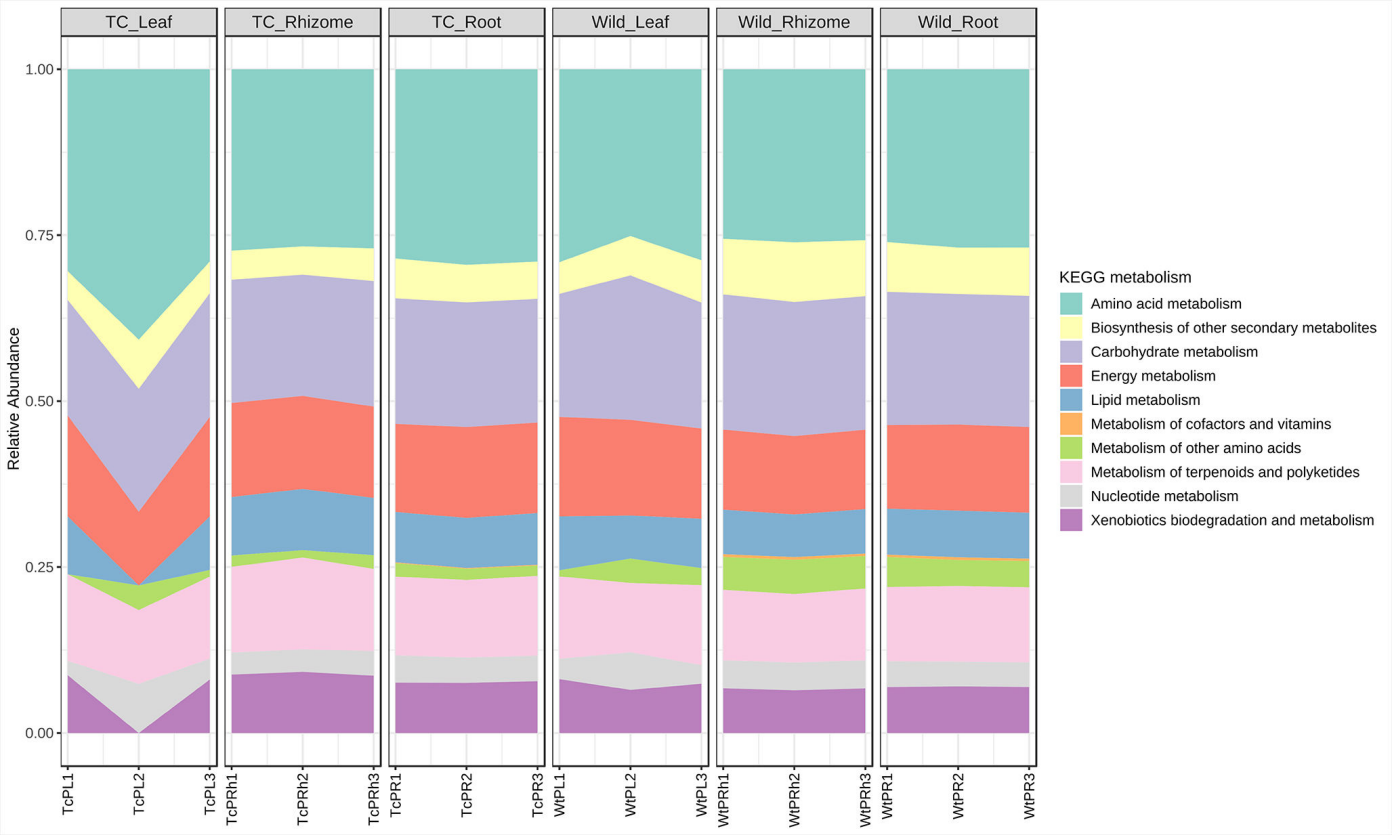
Pathway abundance in the microbial community representing different anatomical parts of Wt and Tc plants.

The predictive functional profiling of the endophytic microbiota of *P. kurrooa* in both the Wt and Tc plants was conducted using PICRUSt2 software. Wherein 16S RNA sequences-based prediction depending on the KEGG pathway database was carried out, and the gene content was identified. The majority of the predicted functional genes of the endophytic bacterial community of *P. kurrooa* were assigned to carbohydrate metabolism, amino acid metabolism, metabolism of terpenoids and polyketides, energy metabolism followed by biosynthesis of secondary metabolites, lipid metabolism, metabolism of other amino acids, nucleotide metabolism, and genes of xenobiotics biodegradation and metabolism (Fig. 10).

**Fig 11:**
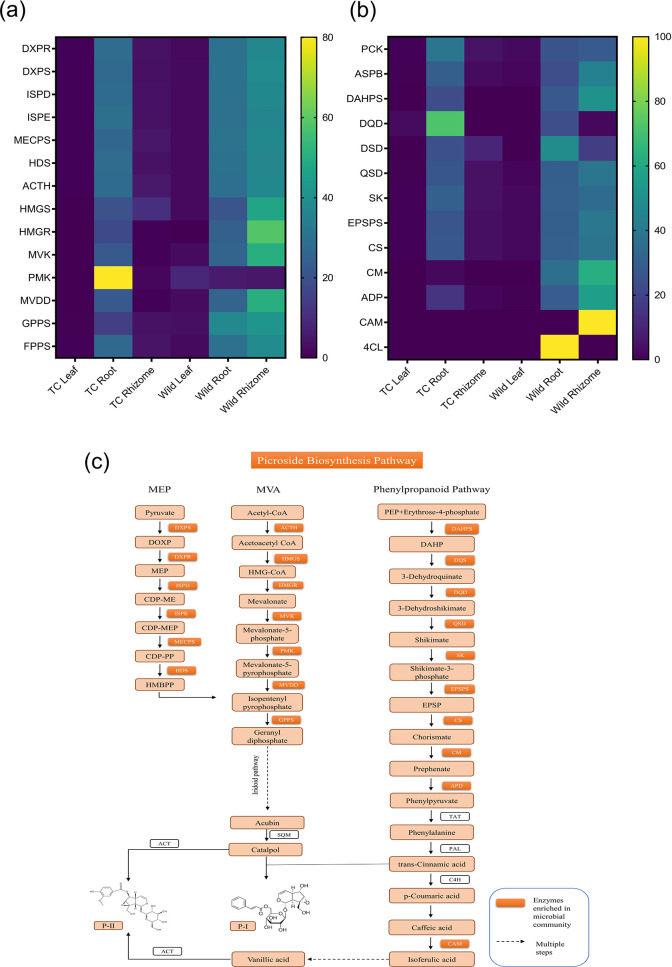
Predictive functional analysis of endophytic bacterial community using PICRUSt2, representing different anatomical parts of Wt and Tc plants. Heat map representing the predicted genes that encode key enzymes involved in picroside biosynthesis; (**a**) Terpenoid backbone (MVA and MEP) pathway; (**b**) Shikimate and Phenylpropanoid Pathway; (**c**) Picrosides biosynthesis pathway with enzymatic genes (red colored box) enriched in the endomicrobial community. DXPR, 1-deoxy-D-xylulose 5-phosphate reductase; DXPS, 1-deoxy-D-xylulose 5-phosphate synthase; ISPD, 2-C- methylerythritol 4-phosphate cytidyl transferase; ISPE, 4-(cytidine-50-diphospho)−2-Cmethylerythritol kinase; MECPS, 2-C-methylerythritol-2,4- cyclophosphate synthase; HDS, 1-hydroxy-2-methyl- 2-(E)-butenyl 4-diphosphate synthase; ACTH, Acetoacetyl-CoA thiolase; HMGS, Hydroxymethyl glutaryl CoA synthase; HMGR, Hydroxymethyl glutaryl CoA reductase; MVK, Mevalonate kinase; PMK, Phosphomevalonate kinase; MVDD, Mevalonate diphosphate decarboxylase; GPPS, Geraniol diphosphate synthase; FPPS, farnesyl-diphosphate synthase; PCK, phosphoenolpyruvate carboxykinase; ASPB, aspartate aminotransferase; DAHPS, 3-Deoxy-D-arabinoheptulosonate 7- phosphate synthase; DQD, Dehydroquinate dehydratase; DSD, Dehydroshikimate dehydratase; QSD, Quinate/shikimate dehydrogenase; SK, Shikimate kinase; EPSPS, 5- enolpyruvylshikimic acid-3-phosphate synthase; CS, Chorismate synthase; CM, ChorIsmate mutase; ADP, Arogenate/prephenate dehydratase; CAM, Caffeic acid-3- o-methyltransferase; 4Cl, 4-coumarate-CoA ligase; TAT, Tyrosine aminotransferase; PAL, Phenylalanine ammonia lyase; C4H, Cinnamate 4 hydroxylase.

Further genes involved in the biosynthesis of secondary metabolites (especially for picrosides biosynthesis) were enriched. The biosynthesis of picrosides is mainly through the mevalonate (MVA), non-mevalonate (MEP), iridoid, and shikimate/phenylpropanoid pathways. We found that the genes involved in terpenoid backbone biosynthesis which is associated with MVA, MEP, and iridoid pathways were present in the endophytic bacterial community, interestingly the presence of these genes was higher in endophytic community associated with different parts of Wt plants when compared to the endophytic community of Tc plant of *P. kurrooa* (Fig. 11a; Table S6). Similarly, genes of the shikimate/phenylpropanoid pathway were also enriched in the endophytic community associated with Wt plants (Fig. 10b; Table S6).

## DISCUSSION

Recent advancements in plant microbiota research have suggested that microorganisms surviving on the outside and inside of plants are significant drivers of host health and development through their synergistic role in enhancing immunity, nutrient acquisition, and tolerance to various abiotic/biotic stresses (7, 45). Domestication of wild plants, industrial processes, breeding, and overuse of pesticides/fertilizers have caused loss of associated microbial diversity from these plants, and reinstating them could improve plant health (46, 47). Disruption of symbiotic interactions between plants and mycorrhiza in domesticated crops resulted in detrimental effects on colonization and decreased response to growth mediated by the fungal symbionts (48). Identification of genes involved in microbial adaptation to plant environment could be possible due to large-scale comparative metagenomics (49). These approaches benefited plants’ microbial rewilding by recognizing essential genes underlying favorable interactions across crop species. The microbiome association in plants consists of three types of interaction, namely host-to-microbe, microbe-to-host, and microbe-to-microbe, having their evolutionary features (50). Transferring complex microbial communities associated with wild plant root and shoot tissue into the seeds/planting material of their cultivated/domesticated counterparts can initially be identified by microbe-associated plant beneficial properties (51). In nature, plants associate and interact with a myriad of microorganisms, referred to as plant microbiome, wherein bacterial and fungal communities live within the plants and considered as endophytic community establishing a non-pathogenic relationship with the host and imparting essential roles in plant health (52) and growth (53 – 58).

### Difference in taxa abundance of bacterial endophytes in different parts of wild and tissue-cultured plants

In the present study, the endophytic microbial community associated with tissue cultured *in-vitro* propagated (Tc) and wild type (Wt) natural plants were explored. Therefore, to achieve this 16S amplicon sequencing was performed from different parts of *P. kurrooa* Wt and Tc plants. As it is well established that the endophytes are present in different parts of plants and also show plant-tissue specificity, therefore different parts of plants were selected for endomicrobiome study. Our analysis revealed that overall endophytic bacterial diversity was less in Tc plants than in Wt plants. The xylem of *in vitro* propagated olive (*Oleo europaea* subsp. *europaea var. europaea*) had decreased total OTU numbers when compared to plants multiplied under non-aseptic conditions (59). Systematically compared endophytic microbial community structure of rice in African and Asian cultivars revealed that wild cultivars had more root endophytes when compared to cultivated rice in their first generation following crossbreeding; also network analysis of bacterial and fungal OTUs showed wild species were able to form clusters with higher significant correlations than cultivated rice fungal species (60). Tissue-specific microbial diversity abundance suggested that the rhizome and roots of both Wt and Tc plants harbor more endophytic bacterial community than leaf tissues, signifying relative and absolute abundances of endophytes are associated with the different parts of the plant. The phyllosphere diversity of *Bourardia ternifolia,* a medicinal plant, is less than root and rhizosphere (61). Microbiome diversity analysis of California citrus orchard also revealed that the overall diversity in above-ground tissues was less than root exosphere and endosphere (62). The predominant phyla found in the different parts of *P. kurrooa* are also known to colonize as endophytic bacterial communities in other plants, including Arabidopsis, Maize, Tamarix, *B. ternifolia,* Seagrass, and Cinnamon (63 – 68). Followed by the most abundant phyla Proteobacteria in all parts of Wt and Tc *P. kurrooa* plants, abundance of phylum Bacteroidetes and OD1 in rhizomes and roots of Wt plants, clearly discriminate the endophytic community of Wt plants from Tc plants where OD1 (Tc rhizome) and Firmicutes (Tc roots) were abundant. Among the distinct endophytic communities harbored by the Wt plant many are already known to associate with diverse plants with beneficial roles in plant growth promotion and production or modulation of biosynthesis of *in planta* secondary metabolites. Endophytic nature of *Paracoccus* has been reported in many plants including medicinal plants such as *Bouvardia ternifolia* (68); it was also isolated from root nodules of *Sphaerophysa salsula* with siderophore-producing property (69). *Massilia* as endophyte was found to be abundant in *Citrus grandis* cv Tomentosa leaves, and species of this genera has been known to synthesize multiple secondary metabolites and enzymes (70). Moreover, it is reported to also have properties such as phosphorus solubilization, degradation of phenanthrene, and heavy metals resistance (71). *Arsenophonus* genus although known as an insect intracellular symbiont (72), it has been also reported as an endophyte in phylloshpere of guarana (*Paullinia cupana*), a Brazilian crop of amazon (73). Endophytic *Acidovorax radices* producing N-acyl homoserine lactone (AHL) influenced plant growth promotion and caused accumulation of several flavonoid compounds such as saponarin and lutanarin (74). *Microbacterium* sp. isolated from the *Catharanthus roseus*, was found to produce host therapeutic important vindoline, a terpenoid indole alkaloids, used to treat Hodgkin’s disease and acute leukaemia (75). *Pseudoxanthomonas* sp. is reported as endophyte in many plants such as pear, peach, *Geum aleppicum,* wheat, rice, and so on (76 – 78). The endophytic nature of *Novosphingobium* has been reported in cacao plants, with anti-fungal volatiles producing properties (79), also its role in growth promotion in rice has already been established (80). *Steroidobacter* genus was found to be the most predominant genera in the root tissue of mulberry (Moris L.) under cold condition (81), *Hydrogenophaga* was found to colonize root tissue of *Lolium perenne* (82) and other crops (83). *Duganella* was found as a core microbiome of wheat Rokosz cultivar (84), and in soil of cultivated maize field (85). *Rhizobium* has been reported as endophyte in many plants (86), with synergistic effect on plant biomass, chlorophyll a content, N fixation and P and Zn solubilization (87). Root-associated *Variovorax* in sunflower promoted plant growth, degradation of xenobiotics and quorum-quenching activity (88). *Rheinheimera* sp. isolated from the rhizospheric soil of the medicinal plant *Echinacea purpurea* helps in root elongation and morphology (89); the genera was first isolated from roots of rice grown in soil (90) and also from rhizosphere of barley (*Hordeum secalinum)* (91). Genus *Pedobacter* has been isolated as endophyte in many plants like maize, *Carex pumila*, rice, wheat, potato with varied plant growth promotion properties (44, 92 – 95). *Luteolibacter* abundance in the rhizosphere and rhizoplane of *Miscanthus x giganteus* and its role in heavy metal-contaminated soil remediation has already been reported (96). Endophytic communities distinctly observed in the rhizome of Wt plants are also found to colonize other plants. A novel *Cellvibrio* sp. from roots of *Zantedeschia aethiopica* is reported (97), with nitrogen-fixing property (98) and also as an enriched endophyte in salinity stress in *Medicago truncatula* (99). *Agrobacterium* genera have been reported as an endophyte in many plants such as *Sesbania cannabina* (100), rose (101), *Phaseolus vulgaris* (102), and *Wisteria sinensis* (103). Similarly, *Flavobacterium* was found as an endophyte in *Suaeda corniculate* (104), *Phragmites australis* (105)*, Panax ginseng* (106)*,* maize (107), rice (108), and soybean (109) with many plant-beneficial properties. Endophytic *Enterobacter* is well established in maize (110), wheat (111), and chickpea (112). *Bradyrhizobium* as an endophyte and its role in enhancing nodulation and photosynthetic ability have been investigated in rice (113 – 115), peanut (116), and soybean (117). *Phaeospirillum* genera as potential nitrogen fixing bacteria was reported in Atacama desert giant horsetail plant (118), and also as an endophyte in wheat (119). These distinct plant-beneficial endophytic communities associated with different parts of wild *P. kurrooa* may be responsible for higher content of secondary metabolites and absence of these communities in Tc plant reduced the secondary metabolites. Studying these distinct endophytic communities may help to understand how the plant-associated microbial community assists and interacts with its host in imparting growth and tolerance toward natural stress, which become lost during *in vitro* propagation of plants, ultimately affecting the overall plant robustness and growth ability.

### Diversity indices across different parts of Wt and Tc plants

Alpha diversity is the diversity within a community utilizing the total number of OTUs (richness), the relative abundance present (Shannon diversity), or indices that combine these two dimensions (evenness). Higher values of richness, Shannon and Simpson index of leaves, roots, and rhizome tissues of Wt plants indicated higher endophytic diversity of these parts compared to different parts of Tc plants. Besides, in Wt plants, the rhizome had the highest alpha diversity and therefore more endophytic diversity than the roots and leaves; however, in Tc plants, roots had the highest alpha diversity than the rhizome and leaves tissues. Several studies have generally used alpha diversity of microbes to investigate the relationships between the functioning of microbial communities and their structure (120, 121). Beta diversity is used most frequently to define the biological diversity composition of microbes along the environmental gradients (122, 123). In our investigation, we observed that the root and rhizome of the Wt and Tc plants formed distinct groups, whereas diversity in the leaf tissue of both the Wt and Tc plant formed close groups suggesting some conserved type endophytic bacterial community in the phylloshpere of both plants. Different studies have also reported that endophytic communities in leaf samples were constant, whereas samples from other tissues such as bark, showed varied composition (124).

### Secondary metabolite profiling and its association with the microbial community of *Picrorhiza kurrooa*

Secondary metabolite profiling revealed that the picrosides and its immediate precursor are relatively low in the Tc plants in comparison to the Wt plants, which has been also demonstrated in previous reports, where *in vitro* cultures generally give around fivefold lower yields of P-I (2, 5, 6, 125). The tissue-specific accumulation of picrosides P-I in leaf and rhizome tissues, and P-II in root and rhizome of both Wt and Tc plants is corroborated with previous studies showing exclusive biosynthesis of P-I in leaves and P-II in root, and accumulation of both in rhizomes (126 – 128). A similar trend was followed by the precursor metabolites of shikimate pathway Cinnamic acid and Vanillic acid. The fate of iridoid pathway precursors catalpol and acubin showed similar accumulation in leaf tissues, but the accumulation of P-I was significantly low in leaves of Tc plants as compared to Wt plants. This observed phenomenon can be explained by considering how the metabolic flux works in picroside biosynthesis; an iridoid glycoside P-I is synthesized by esterification an acyl group (cinnamic acid) to catalpol, which is the iridoid backbone (129). The metabolic basis of biosynthesis of P-I was addressed in the study, wherein through their investigation it was revealed that catalpol and cinnamic acid work in a synergistic manner, and exogenous application of catalpol could only increase the P-I content when cinnamic acid is not in a limiting state, which suggests that both cinnamic acid and catalpol must exist in high amounts for P-I biosynthesis to occur (125). Therefore, although Tc leaf had catalpol and acubin content comparable to Wt leaf, the low content of cinnamic acid could be the contributing factor for low P-I accumulation in Tc leaf.

Further, to understand this phenomenon RDA analysis was executed to relate the picroside content to the bacterial diversity. The RDA has been utilized in different studies to correlate microbial communities and host metabolites (63, 130). It is already a well-established factor that endophytes can alter the secondary metabolism of their host plant or work in association with the plant to complete the biosynthesis of secondary metabolites that ultimately accumulate in the plant. Maytansine biosynthesis in *Maytenus serrata* is found to be orchestrated by its endophytes, a dynamic cross-species interaction in secondary metabolite production (131). There is considerable evidence suggesting qualitative and quantitative variations of pharmaceutically and industrially important secondary metabolites of medicinal plants are influenced by the microbiome (36) *Duganella* species which is solely present in the roots and rhizomes of Wt plants have been known to produce a variety of secondary metabolites (132). The presence of gene clusters of secondary metabolites and type VI gene clusters in *Duganella* species, many of which have antifungal properties, and the production of violacein, a major antibiotic, has been also established in many studies (132 – 135). The association of endophytic *Duganella* with secondary metabolites such as alkaloids and others has previously been also observed in Chinese fir (136). *Bradyrhizobium* is an Alphaproteobacteria that can undergo nitrogen-fixation symbiosis in the roots of many crops (137) found to contain gene clusters responsible for the biosynthesis of terpenes (138), phenolic compounds such as coumestrol (139), and alkaloids such as monocrotaline (140), linking their role in the production of secondary metabolites usually produced by the host. Endophytic *Massilia* genera are known to promote terpenoid content in *C. camphora*, an important medicinal plant in traditional Chinese medicine (130). *Streptococcus* has been established as an endophyte in crops, such as maize (141). The similarity of metabolic pathways such as carbohydrate and amino acid metabolism, metabolism of terpenoids and polyketide between endophytic bacteria and different plants *Cinnamomum camphora* (142), and *P. notoginseng* (143) have been reported, suggesting a close symbiotic association with the host plant. Secondary metabolites regulate plant growth, photosynthesis, signal transduction, along with development and can be enhanced by endophytic bacteria (144).

Endophytic bacterial secondary metabolism can assist the secondary metabolite production of *P. kurrooa,* which may be due to the following factors: Picrosides are monoterpenoids, and there are reports suggesting that both plants and endophytes have shared metabolic processes, and the biosynthesis pathway of terpenoids in endophytic bacteria is also found similar as in plants (145, 146). Also the proportion of enhanced metabolism of terpenoids and polyketides by the endophytic bacteria directly increases the accumulation of terpenoids in the plant; terpenoids play an essential role in photosynthesis, growth, and development, and intracellular signal transduction (144); therefore, the terpenoids produced can synergistically impact plant growth and secondary metabolism. The signal transduction system within the cell is the crucial bridge regulating the association of endophytic bacteria and plant secondary metabolites biosynthesis. Endophytes influence the host metabolites by acting as inducers. During the colonization of endophytes, the plants interact with bacterial molecules which leads to the activation of signaling networks and other biological processes which influence the expression of related genes and mediate the biosynthesis and accumulation of plant secondary metabolites. The responses which generate intracellular signals for the generation of secondary metabolites include the jasmonic acid, salicyclic acid, and hydrogen peroxide signaling (147).

### Potential functional analysis of endophytic bacterial community

PICRUSt2 analysis predicted the functions of the targeted metagenome and showed functional abundances of genes encoding enzymes involved in secondary metabolite biosynthesis in endophytic communities of different parts of Wt plants. Although PICRUSt gives a predictive view of the genes present in the bacterial community, its correlation with spatial dynamics of gene abundance and secondary metabolite accumulation could corroborate our prediction. Enzymes involved in picrosides terpenoids backbone pathway (MVA and MEP pathways) were enriched relatively higher in the endophytic community of Wt plants than Tc plants. Though these genes were present throughout different tissues of Wt and Tc plants, the enrichment was relatively higher in the root and rhizome tissue of Wt plants. The enriched enzymes belonged to MEP and MVA pathways: DXPS, DXPR, ISPD, ISPE, MECPS, HMGS, HMGR, MVK, PMK, MVDD, and GPPS, which are responsible for the biosynthesis of the terpenoid backbone of picrosides. Moreover, Wt plants' root and rhizome tissue also showed higher accumulation of P-I, P-II, and P-III compared to Tc plants. The roles of these enzymes in picrosides biosynthesis has been established in numerous investigation (2, 128, 148 – 151). GPPS is an essential precursor for the biosynthesis of monoterpenes (152) and a branch point enzyme for P-I and P-II synthesis (2, 150, 153). Whereas in the case of shikimate and phenylpropanoid pathways, through which the synthesis of functional group moieties, cinnamate (P-I) and vanillate (P-II) occur. The genes enriched were 3-deoxy-7-phosphoheptulonate synthase (DAHPS) which catalyzes the entry step into the shikimate pathway for picroside biosynthesis (125). Further downstream genes of pathway DQD and SK were also enriched which have a direct correlation to picroside content (2), and CM which is involved in convergent flux toward biosynthesis of picroside (151, 154).

### Limitations and future directions

While numerous strategies have been investigated to enhance the secondary metabolite content in cultivated/*in vitro* propagated plants of *P. kurrooa* to match that of their natural counterparts (5, 155 – 158), there remains an unexplored area of research concerning the characterization of the microbial community present in the natural plants and its potential role in picroside biosynthesis. The abundance of evidence supporting the involvement of endophytic bacteria in the production of secondary metabolites by their host organisms necessitates consideration. Our findings underscore the presence of greater microbial diversity and picroside levels in wild-type (Wt) plants, implying a dynamic interplay between host and microbes responsible for this phenomenon. However, it is important to acknowledge that other factors, such as soil, habitat, and various environmental influences, may also contribute to the higher secondary metabolite content in wild plants (159 – 161), but replicating these complex conditions in cultivation conditions is a significant challenge. Therefore, a promising approach for the development of sustainable, high-quality products involves the identification of potential microbial communities from wild plants and their reintroduction into cultivated plants. Therefore, the outcomes of this research offer valuable insights and pave the way for future developments in metabolome studies, focusing on the profiled endophytic bacterial communities residing within this medicinal plant. These bacterial communities likely play a crucial role in the biosynthesis of therapeutic and bioactive compounds, including picrosides, which are among the key compounds of interest in this species. Despite the acknowledged constraints, this contribution lays the foundation for further investigations into the relatively understudied microbiome of this medicinal plant.

### Conclusion

Thus, our study explored the endophytic microbial community dynamics of the Himalayan medicinal plant *P. kurrooa*, a novel investigation of this important medicinal plant. Comparative endophytic diversity analysis of Wt and Tc plants revealed a loss of microbial diversity in the *in vitro* propagation of the plants. Further, the secondary metabolite profiling corroborated with the microbial diversity, with the Tc plants having a low accumulation of secondary metabolites, whereas accumulation was higher in Wt plants with greater microbial diversity. Additionally, the predictive enrichment of microbial genes and their functional abundance using PICRUSt revealed the abundance of enzymes involved in the picrosides biosynthesis pathway, namely MVA, MEP, and Shikimate/Phenylpropanoid pathways in the associated endophytic bacterial communities, with a higher abundance of these enzymes/genes in the Wt plant. These findings would provide novel insight into the endophytic community associated with *P. kurrooa* plants, and also pave a path for future studies on understanding the biosynthesis pathway and direct the focus on how the microbes could play a role in regulating the biosynthesis of picrosides. Also, a new strategy can be formulated, using endophytic microbes to develop sustainable, high-quality micro-propagated plants for industrial applications.

## MATERIALS AND METHODS

### Plant collection

Wild plants (Wt) of *P. kurrooa* collected from Rohtang pass, Himachal Pradesh (N 32°22′27.12″, E 77°15′21.48″) at an elevation of 3,992 masl. The plants were extracted using a shovel and gardening gloves, with intact roots and rhizomes along with the soil, and placed in sterile bags. The taxonomist at our institute identified the collected plants and submitted them as voucher specimen number PLP16488. For the tissue cultured plants (Tc) population, *in vitro* propagated plants were used, where the explant source was from the wild plants collected. Plants with proper roots and rhizomes were further processed for downstream application. Samples collection for both Wt and Tc plants was done by making a composite sample of 10 plants treated as one replicate, a total of three replicates were processed independently (*n* = 30).

### Sample processing and DNA extraction

DNA was isolated from equal amounts of tissue from different vegetative parts of plants including leaves, roots, and rhizomes of Wt and Tc plants. Tissues were cut into segments of ~3 cm under strict aseptic conditions using sterile scalpel blades. For surface sterilization (to remove any surface microbes), the segments were rinsed three times with sterile double distilled water to remove soil or dust and were then treated with 70% ethanol for 1 min, 2.5% sodium hypochlorite for 5 min, 70% ethanol for 30 s, and rinsed with sterile water five times. Further, to check the efficiency of the surface sterilization process, the water after the final rinse of tissue was spread (400 µL) using a sterile glass rod onto nutrient broth (NB) media plates. The plates were then incubated for 24 h at 28°C. No growth of bacteria was detected in the plates. The surface sterilized plant tissues were crushed under aseptic conditions with the help of sterile pestle and mortar using liquid nitrogen. DNA was extracted from all the tissues using Fast DNA SPIN Kit for Soil (MP Biomedicals, USA), following the manufacturer’s protocol. The quality of DNA was estimated on agarose gel and quantification was done using Bio-spectrophotometer (Eppendorf, Germany) and Qubit fluorimeter (Thermo Fisher Scientific, USA).

The DNA extracted from samples was employed for library preparation through the application of QIAseq 16S/ITS region panels (Qiagen, Germany) following the manufacturer’s instructions. Negative controls, where sterile water substituted the template DNA, were also included. The DNA library’s quality and quantity were assessed using the Bioanalyzer (Agilent Technologies, USA). No amplicons were detected in the negative controls, and they were not subjected to sequencing. Subsequently, the prepared libraries were sequenced using the Illumina MiSeq platform.

### Amplicon-based bioinformatics analysis

The raw reads from Illumina sequencing were demultiplexed using barcodes, and the barcodes were removed; the quality of the reads was checked by FastQC v0.11.9 (https://www.bioinformatics.babraham.ac.uk/projects/fastqc/) and reads with Phred score less than 30 were removed using Cutadapt v3.4 (162). All samples' trimmed reads were loaded into the QIIME2 v2021.2 pipeline for estimating microbial diversity and composition (163). DADA2 was used to remove chimeric sequences from imported reeds after being denoised to give non-chimeric denoised paired-end reads (164). The reads were then classified into OTUs based on Greengenes v13.8 database (165). Multiple sequence alignment was performed using the MAFFT tool on the representative sequences acquired after denoising (166). The alignment was then masked to eliminate highly variable sites.

### Microbial abundance and diversity analysis

Phyloseq R package was utilized to calculate and plot Alpha diversity measures (Observed, Shannon, and Simpson index), whereas Beta diversity analysis was done using the Microbiome Analyst R server (167 – 170). Taxa associated with the plants were filtered and removed manually from the OTU.biom file before analysis. The taxonomic labels and OTU tables generated from QIIME-2 were uploaded to the Microbiome Analyst R server. Additionally, samples with low count features with less than 10% prevalence were removed, and prior to diversity analysis, samples were also rarefied to minimum library size. Microbiome analyst R server was utilized for statistical analysis; for alpha diversity parametric *t* test with analysis of variance (ANOVA) and non-parametric Mann-Whitney test with Krushal-Wallis statistics was utilized to determine the significant difference in alpha diversity indices between different tissues (leaf, root, and rhizome) of Wt and Tc plants. Statistical analysis for Beta-diversity was done using Bray-Cutis dissimilarity matrix and Jaccard distance index after log transformation of rarefied abundance data. To visualize the Beta-diversity, PCoA plots were utilized to understand bacteria community composition differences across different tissues of Wt and Tc plants. PERMANOVA and ANOSIM (analysis of group similarities) were computed to analyze ordination measures between different tissues of Wt and Tc plants for their statistical significance. The Linear discriminant analysis effect size program (LEfSe v1.0) was utilized to analyze the significant difference in the relative abundance of different taxa among different tissues of Wt and Tc plants at a linear discriminant analysis (LDA) cutoff score ≥2 (171). LEfSe employs Krushal-Wallis to identify significantly abundant taxa in different groups, LDA is then applied to the taxa which meet the significance threshold to determine their effect size. To further investigate microbial community which remained unchanged in their composition across the leaf, root, and rhizome tissues of both Wt and Tc, we commuted the core microbiome, two parameters are considered while performing core microbiome; the first one is sample prevalence which is the minimum fraction (percentage) of samples the taxa must be observed in and the other is relative abundance (fraction) of taxa which is taken to consider the taxa as part of core member. Heat tree analysis was also performed, which provides a hierarchical structure of taxonomic classification by quantitative, using median abundance, and statistical by using the non-parametric Wilcoxon rank-sum test, to depict taxonomic differences between Wt and Tc plants (172). LDA, core microbiome, and heat tree analysis and plots were generated using the Microbiome Analyst R server (173).

### UHPLC estimation and correlation with microbial community

Leaves, roots, and rhizomes samples were collected from Wt and Tc plants and used for secondary metabolite profiling. Samples were dried and powdered finely using mortar and pestle, and 100 mg of the powder was macerated in 5 mL HPLC-grade absolute methanol for 24 h. Further samples were sonicated for 30 min at 35°C followed by centrifugation at 1,000 rpm for 2 min. The supernatant was filtered through a 0.22-µm filter and used for ultra-high-performance liquid chromatography (UHPLC-PDA) (174). P-I, P-II, P-III, caffeic acid, cinnamic acid, vanillic acid, catalpol, and aucubin were quantified using UHPLC-PDA (Shimadzu LC-MS 2020) (175). About 1 µL of each sample was injected into Waters BEH C18 column, 1.7 µL (2.1 × 100 mm^2^) coupled to a PDA detector. 0.1% formic acid in water (Solvent A) and 0.1% formic acid in acetonitrile (Solvent B) were used for gradient elution at a constant flow of 0.28 mL/min. Linear gradient (0, 95:5), (9, 65:35), (11, 60:40), (12, 10:90), (16, 10:90), (16.5, 95: 5), and (20, 95: 5) of solvent A and B [vol/vol proportions; t (min), % A:B] was applied. For statistical analysis, the univariate one-way ANOVA with Duncan post hoc multiple comparison tests (*P* ≤ 0.05) was performed. RDA was used to correlate secondary metabolites and bacterial community composition abundancy (63), and RDA was performed using CANOCO software (176).

### Amplicon-based functional prediction of microbial communities

To predict the metabolic pathways associated with the bacterial communities, Phylogenetic investigation of communities by reconstruction of unobserved state (PICRUSt2) software was utilized (177). Based on the database of Kyoto Encyclopedia of Genes and Genome (KEGG) and evolutionary genealogy of genes (EggNOG), the KEGG orthology (KO) was predicted based on the OTU matrix, along with abundance was also obtained. EC number involved in the possible secondary metabolite biosynthesis was manually categorized based on the KEGG database.

## Data Availability

The data of Picrorhiza kurrooa amplicon metagenome sequences generated in the present study have been deposited at GenBank, NCBI repository, under BioProject accession number PRJNA948802.

## SUPPLEMENTAL MATERIAL

Tables S1 to S5 spectrum.02279-23-s0001.docx Table S1 Abundance of different taxa at phylum level present in various anatomical parts of Wt and Tc plants. Table S2 Abundance of different taxa at genus level present in various anatomical parts of Wt and Tc plants. Table S3 Core microbiota of endophytes in different anatomical parts of Picrorhiza kurroa in Wt and Tc plant. Table S4 LEfSe analysis statistical data for each taxon by sample groups for Figure 6. Table S5 Secondary metabolite accumulation (µg/mg DW) in different tissues of Wt and Tc plants of P. kurrooa.

Table S6 spectrum.02279-23-s0002.xlsx Picroside biosynthesis genes enriched in microbial community of P. Kurrooa

ASM does not own the copyrights to Supplemental Material that may be linked to, or accessed through, an article. The authors have granted ASM a non-exclusive, world-wide license to publish the Supplemental Material files. Please contact the corresponding author directly for reuse.
