# Supplementary material for: Endomicrobiome of in vitro and natural plants deciphering the endophytes-associated secondary metabolite biosynthesis in Picrorhiza kurrooa, a Himalayan medicinal herb
Source: Microbiol Spectr. 2023 Oct 9;11(6):e02279-23. doi: 10.1128/spectrum.02279-23 (PMC10715050; doi:10.1128/spectrum.02279-23)
Supplement: Tables S1 to S5 — Table S1 Abundance of different taxa at phylum level present in various anatomical parts of Wt and Tc plants. Table S2 Abundance of different taxa at genus level present in various anatomical parts of Wt and Tc plants. Table S3 Core microbiota of endophytes in different anatomical parts of Picrorhiza kurroa in Wt and Tc plant. Table S4 LEfSe analysis statistical data for each taxon by sample groups for Figure 6. Table S5 Secondary metabolite accumulation (µg/mg DW) in different tissues of Wt and Tc plants of P. kurrooa. [file spectrum.02279-23-s0001.docx]

**Endomicrobiome of *in vitro* and natural plants deciphering the endophytes-associated secondary metabolite biosynthesis in *Picrorhiza kurrooa*, a Himalayan medicinal herb**

Anish Tamang^a,b^, Mohit Swarnkar^a^, Pawan Kumar^c^, Dinesh Kumar^b,c^, Shiv Shanker Pandey^a,b^#, and Vipin Hallan^a,b^#

^a^Biotechnology Division, CSIR-Institute of Himalayan Bioresource Technology (IHBT), Palampur- 176061 (H.P), India.

^b^Academy of Scientific and Innovative Research (AcSIR), Ghaziabad- 201002, India.

^c^Chemical Technology Division, CSIR-Institute of Himalayan Bioresource Technology, Palampur, 176061, Himachal Pradesh, India.

**# Address correspondence to: Vipin Hallan, E-mail: hallan@ihbt.res.in**

**Shiv Shanker Pandey, E-mail: shivpandey@ihbt.res.in**

**Supplementary Information**

**Table S1: Abundance of different taxa at phylum level present at various anatomical parts of Wt and Tc plants**

| Taxonomy | Tc Leaf | Tc Root | Tc Rhizome | Wt Leaf | Wt Root | Wt Rhizome |
| --- | --- | --- | --- | --- | --- | --- |
| p__Proteobacteria | 47 | 5072 | 745 | 312 | 6488 | 6777 |
| p__Bacteroidetes | 0 | 23 | 0 | 0 | 521 | 926 |
| p__Spirochaetes | 0 | 0 | 8 | 0 | 15 | 12 |
| p__OD1 | 0 | 46 | 127 | 0 | 454 | 471 |
| p__Verrucomicrobia | 0 | 0 | 0 | 0 | 118 | 0 |
| p__Actinobacteria | 0 | 50 | 0 | 0 | 34 | 0 |
| p__Firmicutes | 0 | 329 | 0 | 0 | 12 | 10 |
| p__TM6 | 0 | 0 | 0 | 0 | 0 | 111 |

**Table S2: Abundance of different taxa at genus level present at various anatomical parts of Wt and Tc plants**

| **Taxonomy** | **Tc Leaf** | **Tc Root** | **Tc Rhizome** | **Wt Leaf** | **Wt Root** | **Wt Rhizome** |
| --- | --- | --- | --- | --- | --- | --- |
| g__Acidovorax | 0 | 0 | 0 | 0 | 151 | 0 |
| g__Variovorax | 0 | 12 | 0 | 0 | 80 | 202 |
| f__Oxalobacteraceae | 0 | 146 | 0 | 0 | 0 | 466 |
| g__Arsenophonus | 0 | 0 | 0 | 11 | 0 | 0 |
| g__Polaromonas | 0 | 2 | 2 | 0 | 0 | 24 |
| f__Bradyrhizobiaceae | 0 | 0 | 49 | 0 | 0 | 131 |
| g__Steroidobacter | 0 | 0 | 51 | 0 | 48 | 27 |
| g__Dyadobacter | 0 | 0 | 0 | 0 | 0 | 20 |
| g__Brevundimonas | 0 | 44 | 0 | 0 | 0 | 0 |
| f__Rhizobiaceae | 0 | 216 | 0 | 0 | 144 | 938 |
| p__Proteobacteria | 0 | 130 | 15 | 0 | 589 | 82 |
| g__Flavobacterium | 0 | 23 | 0 | 0 | 310 | 653 |
| g__Bradyrhizobium | 0 | 0 | 0 | 0 | 0 | 62 |
| g__Pseudomonas | 0 | 164 | 73 | 0 | 300 | 273 |
| g__Acinetobacter | 29 | 2494 | 234 | 188 | 583 | 622 |
| g__Agrobacterium | 0 | 82 | 0 | 0 | 368 | 805 |
| g__Ralstonia | 0 | 894 | 50 | 0 | 213 | 61 |
| f__Spirochaetaceae | 0 | 0 | 8 | 0 | 15 | 0 |
| f__Methylophilaceae | 0 | 0 | 0 | 0 | 382 | 343 |
| p__OD1 | 0 | 23 | 0 | 0 | 191 | 279 |
| g__Cellvibrio | 0 | 36 | 0 | 0 | 1160 | 637 |
| f__Caulobacteraceae | 0 | 0 | 0 | 0 | 0 | 85 |
| f__Cytophagaceae | 0 | 0 | 0 | 0 | 201 | 237 |
| g__Luteolibacter | 0 | 0 | 0 | 0 | 85 | 0 |
| f__Methylophilaceae | 0 | 0 | 0 | 0 | 96 | 56 |
| g__Microbacterium | 0 | 0 | 0 | 0 | 21 | 0 |
| g__Desemzia | 0 | 102 | 0 | 0 | 0 | 0 |
| g__Bacillus | 0 | 40 | 0 | 0 | 0 | 0 |
| g__Paracoccus | 0 | 274 | 9 | 8 | 15 | 27 |
| g__Aerococcus | 0 | 177 | 0 | 0 | 12 | 10 |
| g__Pseudoxanthomonas | 0 | 0 | 0 | 0 | 11 | 0 |
| g__Pedomicrobium | 0 | 0 | 0 | 0 | 0 | 26 |
| c__SJA-4 | 0 | 0 | 0 | 0 | 0 | 111 |
| g__Rheinheimera | 0 | 0 | 0 | 0 | 583 | 30 |
| f__Enterobacteriaceae | 0 | 202 | 0 | 0 | 0 | 27 |
| f__Sinobacteraceae | 0 | 0 | 66 | 0 | 0 | 99 |
| f__Comamonadaceae | 0 | 27 | 0 | 0 | 462 | 19 |
| g__Candidatus Portiera | 4 | 0 | 0 | 100 | 0 | 0 |
| g__Devosia | 0 | 0 | 0 | 0 | 0 | 45 |
| f__Hyphomicrobiaceae | 0 | 0 | 0 | 0 | 0 | 158 |
| g__Phaeospirillum | 0 | 0 | 0 | 0 | 0 | 112 |
| g__Burkholderia | 0 | 82 | 0 | 0 | 0 | 0 |
| g__Novosphingobium | 0 | 0 | 0 | 0 | 24 | 0 |
| g__Enterobacter | 0 | 231 | 0 | 0 | 0 | 73 |
| g__Rhizobium | 0 | 0 | 0 | 0 | 564 | 13 |
| g__Hydrogenophaga | 0 | 0 | 16 | 0 | 115 | 15 |
| g__Streptomyces | 0 | 0 | 0 | 0 | 13 | 0 |
| g__Duganella | 0 | 0 | 0 | 0 | 225 | 787 |
| f__Rickettsiaceae | 0 | 0 | 0 | 0 | 0 | 84 |
| g__Aquamonas | 0 | 0 | 182 | 0 | 300 | 419 |
| g__Peredibacter | 0 | 0 | 0 | 0 | 33 | 0 |
| g__Neisseria | 14 | 0 | 0 | 0 | 0 | 0 |
| g__Propionibacterium | 0 | 50 | 0 | 0 | 0 | 0 |
| f__Verrucomicrobiaceae | 0 | 0 | 0 | 0 | 33 | 0 |
| g__SC3-56 | 0 | 0 | 0 | 0 | 10 | 0 |
| g__Asticcacaulis | 0 | 0 | 0 | 0 | 13 | 0 |
| f__Comamonadaceae | 0 | 0 | 0 | 0 | 0 | 2 |
| f__Bacillaceae | 0 | 10 | 0 | 0 | 0 | 0 |
| g__Ochrobactrum | 0 | 29 | 0 | 0 | 0 | 0 |
| o__Rickettsiales | 0 | 0 | 0 | 0 | 18 | 26 |
| p__OD1 | 0 | 23 | 127 | 0 | 263 | 192 |
| g__Massilia | 0 | 0 | 0 | 16 | 0 | 0 |
| g__Pedobacter | 0 | 0 | 0 | 0 | 0 | 16 |
| o__Myxococcales | 0 | 0 | 0 | 0 | 0 | 25 |
| g__Delftia | 0 | 9 | 0 | 0 | 0 | 0 |
| g__Legionella | 0 | 0 | 0 | 0 | 11 | 0 |
| g__Spirochaeta | 0 | 0 | 0 | 0 | 0 | 12 |

**Table S3: LEfSe analysis statistical data for each taxon by sample groups for Figure 6**

| Taxa | Pvalues | FDR | TC_Leaf | TC_Rhizome | TC_Root | Wild_Leaf | Wild_Rhizome | Wild_Root | LDAscore |
| --- | --- | --- | --- | --- | --- | --- | --- | --- | --- |
| Cellvibrio | 0.004893 | 0.030349 | 0 | 0 | 0 | 0 | 905800 | 1413000 | 5.85 |
| Rhizobium | 0.004938 | 0.030349 | 0 | 0 | 144930 | 0 | 4021700 | 1811600 | 6.3 |
| Neorhizobium | 0.005087 | 0.030349 | 0 | 0 | 0 | 0 | 289860 | 797100 | 5.6 |
| Curvibacter | 0.00675 | 0.030349 | 0 | 797100 | 0 | 0 | 253620 | 652170 | 5.6 |
| Acinetobacter | 0.007683 | 0.030349 | 2391300 | 4239100 | 7391300 | 6739100 | 1702900 | 2500000 | 6.45 |
| Flavobacterium | 0.009048 | 0.030349 | 0 | 0 | 36232 | 0 | 362320 | 253620 | 5.26 |
| Ambiguous_taxa | 0.011476 | 0.030349 | 0 | 579710 | 0 | 0 | 108700 | 0 | 5.46 |
| Rheinheimera | 0.011476 | 0.030349 | 0 | 0 | 0 | 0 | 108700 | 1159400 | 5.76 |
| Enterobacter | 0.01169 | 0.030349 | 2029000 | 833330 | 72464 | 1087000 | 72464 | 144930 | 5.99 |
| Candidatus_Profftella | 0.012672 | 0.030349 | 4782600 | 1992800 | 144930 | 1739100 | 144930 | 108700 | 6.37 |
| Paracoccus | 0.012961 | 0.030349 | 36232 | 326090 | 144930 | 362320 | 0 | 0 | 5.26 |
| Acidibacter | 0.013176 | 0.030349 | 0 | 253620 | 0 | 0 | 36232 | 0 | 5.1 |
| Methylobacillus | 0.014091 | 0.030349 | 0 | 0 | 0 | 0 | 144930 | 434780 | 5.34 |
| Hydrogenophaga | 0.015851 | 0.031701 | 0 | 434780 | 0 | 0 | 36232 | 0 | 5.34 |
| Limnohabitans | 0.019078 | 0.033787 | 0 | 0 | 0 | 0 | 289860 | 72464 | 5.16 |
| Massilia | 0.019307 | 0.033787 | 0 | 0 | 72464 | 0 | 905800 | 72464 | 5.66 |
| Ralstonia | 0.021577 | 0.035539 | 760870 | 398550 | 1666700 | 72464 | 72464 | 181160 | 5.9 |
| Desemzia | 0.045157 | 0.070244 | 0 | 72464 | 108700 | 0 | 0 | 36232 | 4.74 |
| OM43_clade | 0.057156 | 0.072244 | 0 | 0 | 36232 | 0 | 108700 | 36232 | 4.74 |
| Uncultured | 0.059343 | 0.072244 | 0 | 72464 | 0 | 0 | 0 | 0 | 4.56 |
| Pedobacter | 0.059343 | 0.072244 | 0 | 0 | 0 | 0 | 72464 | 0 | 4.56 |
| Dyadobacter | 0.059343 | 0.072244 | 0 | 0 | 0 | 0 | 72464 | 0 | 4.56 |
| Aerococcus | 0.059343 | 0.072244 | 0 | 0 | 72464 | 0 | 0 | 0 | 4.56 |
| Ohtaekwangia | 0.08596 | 0.10029 | 0 | 0 | 0 | 0 | 72464 | 108700 | 4.74 |
| Acidovorax | 0.13405 | 0.15013 | 0 | 0 | 0 | 0 | 36232 | 144930 | 4.86 |
| Ensifer | 0.18066 | 0.19456 | 0 | 0 | 72464 | 0 | 72464 | 36232 | 4.56 |
| Caulobacter | 0.41588 | 0.43128 | 0 | 0 | 0 | 0 | 72464 | 0 | 4.56 |
| Devosia | 0.63857 | 0.63857 | 0 | 0 | 36232 | 0 | 36232 | 36232 | 4.26 |

**Table S4: Core microbiota of endophytes in different anatomical parts of *Picrorhiza kurroa* in Wt and Tc plant.**

| **Taxa** | **Tc Leaf** | **Tc Rhizome** | **Tc Root** | **Wild Leaf** | **Wild Rhizome** | **Wild Root** |
| --- | --- | --- | --- | --- | --- | --- |
| Acidibacter | 0.00 | 1.00 | 0.00 | 0.00 | 0.33 | 0.00 |
| Acidovorax | 0.00 | 0.00 | 0.00 | 0.00 | 0.33 | 1.00 |
| Acinetobacter | 1.00 | 1.00 | 1.00 | 1.00 | 1.00 | 1.00 |
| Aerococcus | 0.00 | 0.00 | 0.67 | 0.00 | 0.33 | 0.00 |
| Rhizobium | 0.00 | 0.67 | 1.00 | 0.00 | 1.00 | 1.00 |
| Ambiguous_taxa | 0.00 | 1.00 | 0.33 | 0.00 | 0.33 | 0.00 |
| Arsenophonus | 0.00 | 0.00 | 0.00 | 0.67 | 0.00 | 0.00 |
| Bradyrhizobium | 0.00 | 0.67 | 0.00 | 0.00 | 0.33 | 0.00 |
| Brevundimonas | 0.00 | 0.00 | 0.33 | 0.00 | 0.00 | 0.00 |
| Candidatus_Portiera | 0.67 | 0.00 | 0.00 | 1.00 | 0.00 | 0.00 |
| Candidatus_Profftella | 1.00 | 1.00 | 0.67 | 1.00 | 1.00 | 0.67 |
| Cellvibrio | 0.00 | 0.00 | 0.67 | 0.00 | 1.00 | 1.00 |
| Curvibacter | 0.00 | 1.00 | 0.33 | 0.00 | 0.67 | 1.00 |
| Cutibacterium | 0.33 | 0.00 | 0.67 | 0.33 | 0.00 | 0.00 |
| Desemzia | 0.00 | 0.33 | 0.67 | 0.33 | 0.00 | 0.33 |
| Devosia | 0.00 | 0.00 | 0.00 | 0.00 | 1.00 | 0.00 |
| Dolosigranulum | 0.33 | 0.00 | 0.00 | 0.00 | 0.00 | 0.00 |
| Dyadobacter | 0.00 | 0.00 | 0.00 | 0.00 | 0.67 | 0.33 |
| Ensifer | 0.00 | 0.00 | 0.33 | 0.00 | 0.67 | 0.00 |
| Enterobacter | 1.00 | 1.00 | 0.67 | 1.00 | 0.67 | 1.00 |
| Flavobacterium | 0.00 | 0.00 | 0.33 | 0.00 | 1.00 | 1.00 |
| Hydrogenophaga | 0.00 | 1.00 | 0.00 | 0.00 | 0.00 | 0.33 |
| Klebsiella | 0.00 | 0.67 | 0.00 | 0.00 | 0.33 | 0.00 |
| Limnohabitans | 0.00 | 0.00 | 0.00 | 0.00 | 1.00 | 0.00 |
| Luteolibacter | 0.00 | 0.00 | 0.00 | 0.00 | 0.00 | 0.67 |
| Massilia | 0.00 | 0.00 | 0.33 | 0.33 | 1.00 | 1.00 |
| Methylobacillus | 0.00 | 0.00 | 0.00 | 0.00 | 0.00 | 1.00 |
| Methylotenera | 0.00 | 0.00 | 0.00 | 0.00 | 0.67 | 0.00 |
| Neorhizobium | 0.00 | 0.00 | 0.33 | 0.00 | 1.00 | 1.00 |
| Nitrobacter | 0.00 | 0.33 | 0.00 | 0.00 | 0.33 | 0.00 |
| Nocardia | 0.00 | 0.00 | 0.00 | 0.00 | 0.33 | 0.00 |
| Ochrobactrum | 0.00 | 0.00 | 0.33 | 0.00 | 0.00 | 0.00 |
| Ohtaekwangia | 0.00 | 0.00 | 0.00 | 0.00 | 0.33 | 0.33 |
| OM43_clade | 0.00 | 0.00 | 0.00 | 0.00 | 0.33 | 0.33 |
| Paracoccus | 0.00 | 0.67 | 1.00 | 1.00 | 0.67 | 0.33 |
| Polaromonas | 0.00 | 0.33 | 0.00 | 0.00 | 0.33 | 0.00 |
| Pseudomonas | 0.00 | 0.00 | 0.00 | 0.00 | 0.67 | 0.67 |
| Ralstonia | 1.00 | 1.00 | 1.00 | 0.00 | 0.33 | 1.00 |
| Rheinheimera | 0.00 | 0.00 | 0.00 | 0.00 | 0.67 | 1.00 |
| Rhodomicrobium | 0.00 | 0.00 | 0.00 | 0.00 | 0.67 | 0.00 |
| Turicibacter | 0.33 | 0.33 | 0.00 | 0.00 | 0.00 | 0.33 |
| Uncultured | 0.00 | 0.67 | 0.00 | 0.00 | 0.33 | 0.67 |

|  | P-I | P-II | P-III | Cinnamic acid | Vanillic acid | Caffeic acid | Acubin | Catalpol |
| --- | --- | --- | --- | --- | --- | --- | --- | --- |
| Wt Leaf | 84.94 ± 0.90a | NQ | NQ | 0.78 ± 0.01a | 0.29 ± 0.00d | 0.21 ± 0.00b | 17.84 ± 0.14a | 18.92 ± 0.52a |
| Wt Root | NQ | 3.32 ± 0.00b | 0.16 ± 0.00c | NQ | 1.23 ± 0.01c | 0.19 ± 0.00bc | NQ | NQ |
| Wt Rhizome | 17.86 ± 0.10b | 44.29 ± 0.53a | 2.74 ± 0.00a | NQ | 18.22 ± 0.018a | 0.87 ± 0.01a | 0.75 ± 0.00c | 3.66 ± 0.19c |
| Tc Leaf | 8.77 ± 0.06c | NQ | NQ | 0.08 ± 0.00b | 0.32 ± 0.00d | 0.19 ± 0.00c | 18.96 ± 0.05b | 13.97 ± 0.54b |
| Tc Root | 0.05 ± 0.00e | 0.24 ± 0.00c | 0.03 ± 0.00d | NQ | 0.08 ± 0.00d | 0.16 ± 0.00d | NQ | NQ |
| Tc Rhizome | 5.63 ± 0.01d | 0.25 ± 0.00c | 0.22 ± 0.00b | 0.02 ± 0.00c | 2.97 ± 0.01b | 0.17 ± 0.00d | NQ | NQ |

**Table S5: Secondary metabolite accumulation (µg/mg DW) in different tissues of Wt and Tc plants of *P. kurrooa*.**

Data presented as mean ± standard error (SE), NQ: not quantified, DW: dry weight. The different letters in the column indicated the statistically significant difference between the mean (p< 0.05, Duncan multiple range test)
